# A Customized At-Home Stool Collection Protocol for Use in Microbiome Studies Conducted in Cancer Patient Populations

**DOI:** 10.1007/s00248-019-01346-2

**Published:** 2019-03-30

**Authors:** Stephanie R. Hogue, Maria F. Gomez, Wildson Vieira da Silva, Christine M. Pierce

**Affiliations:** 1 Department of Cancer Epidemiology, Moffitt Cancer Center and Research Institute, Tampa, FL 33612 USA; 2 Center for Immunization and Infection Research in Cancer, Moffitt Cancer Center and Research Institute, Tampa, FL 33612 USA

**Keywords:** Microbiome, Stool collection, RNAlater, Ethanol, FOBT card

## Abstract

Fecal specimen collection in the clinical setting is often unfeasible for large population studies, especially because cancer patients on immunotherapy often experience constipation. A method for constructing and using an at-home stool collection kit designed for epidemiological studies in cancer patients is presented. Participation and compliance rates of the collection kit among late-stage cancer patients from an ongoing, longitudinal study are also discussed. The kit includes three different media on which samples are introduced. Using one stool sample, patients collect specimens by smearing stool onto a fecal occult blood test (FOBT) card, containing three slides for collection. Additional specimens from the same stool sample are added to one tube containing 8 mL of RNAlater preservative and one tube containing 8 mL of 95% ethanol. Stool specimens are stored at room temperature and returned to researchers within 3 days of collection. The purpose of this kit is to yield stool specimens on a variety of media that can be preserved for extended periods of time at room temperature and are compatible with multi-omics approaches for specimen analysis. According to leading microbiome researchers and published literature, each collection method is considered optimal for use in large epidemiological studies. Moreover, the kit is comprised of various components that make stool collection easy, so as not to burden the patient and hence maximize overall compliance. Use of this kit in a study of late-stage lung cancer patients had a participation rate of 83% and baseline compliance rate of 58%.

**Electronic supplementary material:**

The online version of this article (10.1007/s00248-019-01346-2) contains supplementary material, which is available to authorized users.

## Introduction

The microbiome, the vast collection of microbes inhabiting the human body, has been associated with cancer development and progression [1–4], as well as response to chemotherapy and immunotherapy [5–7], yet the biological mechanisms underlying these associations remain unknown. Prospective epidemiological studies are needed to elucidate these mechanisms and determine the microbiome’s clinical utility—as a biomarker of disease and prognosis and to enhance therapeutic outcomes. However, gut microbiome studies should utilize valid, reproducible, and standardized methods to enhance data comparability across studies, as differences in stool collection methods contribute to inter-study variability [8–10]. Collection using the “gold standard”—immediately freezing stool at − 80 °C or in liquid nitrogen (LN)—is often not feasible in large, human studies. Since most people cannot provide a stool sample when convenient for researchers, stool must be self-collected and refrigerated or stored at room temperature until specimens can be transported to the laboratory. Storing stool specimens in home refrigerators/freezers is not recommended due to automatic defrost cycles which can damage the microbial composition of the sample as it thaws [11]. For specimens stored at room temperature, preservatives must be used to stabilize nucleic acids or other small molecules needed for downstream analyses and should be compatible with multiple omics approaches, including metagenomics (i.e., microbial composition), metatranscriptomics (i.e., microbial function), and metabolomics (i.e., metabolite production).

Here, we describe an at-home stool collection protocol for use in epidemiological studies of the gut microbiome, customized for use among cancer patient populations. We also discuss kit acceptability and use within an ongoing, longitudinal study of late-stage cancer patients. Methods used in this protocol have been previously evaluated by leading microbiome scientists for validity and reproducibility by comparing each method to stool immediately frozen at − 80 °C or in LN without preservatives. Stool collected on an inexpensive filter paper matrix (e.g., fecal occult blood test [FOBT] card or FTA card) adequately maintains microbial signatures and yields similar abundance and diversity measures [10–13] for 16S rRNA gene sequencing; DNA remains stable up to 8 weeks at room temperature before freezing [11]. RNAlater is the most widely recommended preservative for metatranscriptomic studies [14], as it stabilizes RNA up to 6 days without freezing [15]. Ninety-five percent ethanol is recommended for fecal metabolomic studies, as it adequately preserves metabolite signatures when stored up to 4 days at room temperature [16]. The stool collection kit described below integrates each of the above validated methods to preserve stool using standardized methods that are compatible with multi-omics approaches.

## Protocol

Aseptic technique should be utilized during kit assembly to minimize laboratory-introduced contamination. Wear gloves and a lab coat, and disinfect work surfaces with 70% ethanol [17]. Assembling collection tubes in a biological safety cabinet (class II+) is recommended [18]. Avoid talking, coughing, or sneezing to prevent kit contamination. Should the collection spoons, FOBT card windows, or pipettes come into direct contact with any surface, discard them. A list of materials (Table 1) and video tutorial (Online Resource 1) are provided.

**Table 1:** List of materials, including vendor and catalog numbers, used to assemble the at-home stool collection kit. Materials used to store stool specimens are also included

Item name	Vendor	Catalog number	Comments/description	Bulk price^a^	Price per kit
15-mL Sarstedt stool collection tubes with spatulas	Sarstedt (Nümbrecht, Germany)	80.623.022	2 per kit (2 tubes and 2 small plastic spatulas)	$73.30	$0.59
Hemoccult II SENSA triple slide FOBT card	Beckman Coulter (through Fisher Scientific)	SK-64130	Includes the collection card, biohazard envelope, and wooden applicators	$348.68	$2.18
95% (wt/wt) ethanol	N/A	N/A		$17.25	$0.04
RNAlater solution	Invitrogen (through Fisher Scientific)	AM7021	500-mL bottle provides enough RNAlater to produce about 62 kits	$433.67	$6.94
Protocult stool collection device	Protocult (through Fisher Scientific)	NC0441080	2 per kit; individually wrapped without instructions	$138.87	$2.78
Absorbent Pad Mats (18 × 20”)	VWR International	89126-794	1 per kit	$145.86	$0.42
Gloves, medium nitrile	VWR International	82026-426	1 pair per kit	$5.65–5.82	$0.11
Styrofoam tube rack	Sarstedt (Nümbrecht, Germany)	95.064.251	Divide into two 25-tube racks	$35.90	$0.75
10 in. × 12 in. plastic zip bag, 2 mil	RD Plastics (through Fisher Scientific)	23-700-215	To hold rack and tubes	$35.61	$0.06
6 in. × 8 in. plastic zip bag, 2 mil	RD Plastics (through Fisher Scientific)	23-700-214	To hold pad, gloves, and plastic spatulas	$26.20	$0.03
5 3/8 in. × 5 3/8 in. × 6 in. Hazmat shipping box	ULINE	S-7335		$190.00	$1.90
Absorbent pad (3 × 4”)	VWR	89170-926	Place in 10 × 12” zip bag with tubes to absorb any spills—IATA shipping requirement	$53.35	$0.05
Return tape strips, 2 in. × 23 in.	Tape Solutions	150RT2X23	Use if specimens are to be returned by courier	$33.50	$0.34
10 in. × 13 in. frosted shopper bags	Promotions Now	BA1035WE	Choose any color	$1011.12	$1.01
Avery 5160 labels, 1 in. × 2 5/8 in.	Mister Paper	364364	To label 15-mL tubes	$12.51	$0.01
4 in. × 6 in. plastic zip bag, 4 mil	ULINE	S-1302	To store FOBT card at − 80 °C	$53.30	$0.05
7 × 7 storage box	Sarstedt (Nümbrecht, Germany)	95.064.922	To store 15-mL tubes and FOBT cards at − 80 °C	$26.50	$0.11
Total price of one kit					$17.37

^a^Prices may vary based on institutional discounts


ESM 1(MP4 60,542 kb)


### Fecal Collection Tubes

Label a 15-mL Sarstedt collection tube with the preservative type (e.g., 95% ethanol), lot number, and expiration date; leave room on the label for patient ID and study visit ID. In a biological safety cabinet, remove the cap and add 8 mL of 95% ethanol (wt/wt) using a sterile, serological pipette. Close securely and set aside. Repeat the above step, adding 8 mL of RNAlater to a properly labeled tube. Place one 95% ethanol-filled tube and one RNAlater-filled tube into a small Styrofoam rack. Set the rack inside a large (10 in. × 12 in.) zip bag with small absorbent pad and seal.

### Fecal Collection Card

Remove the FOBT card and wooden applicators from the outer envelope and place into the biohazard envelope. Discard the outer envelope and tissue paper.

### Collection Supplies

Place a pair of medium size nitrile gloves, a folded absorbent pad, and two Sarstedt spatulas into a medium (6 in × 8 in) zip bag and seal.

### Kit Assembly

**Fig. 1:**
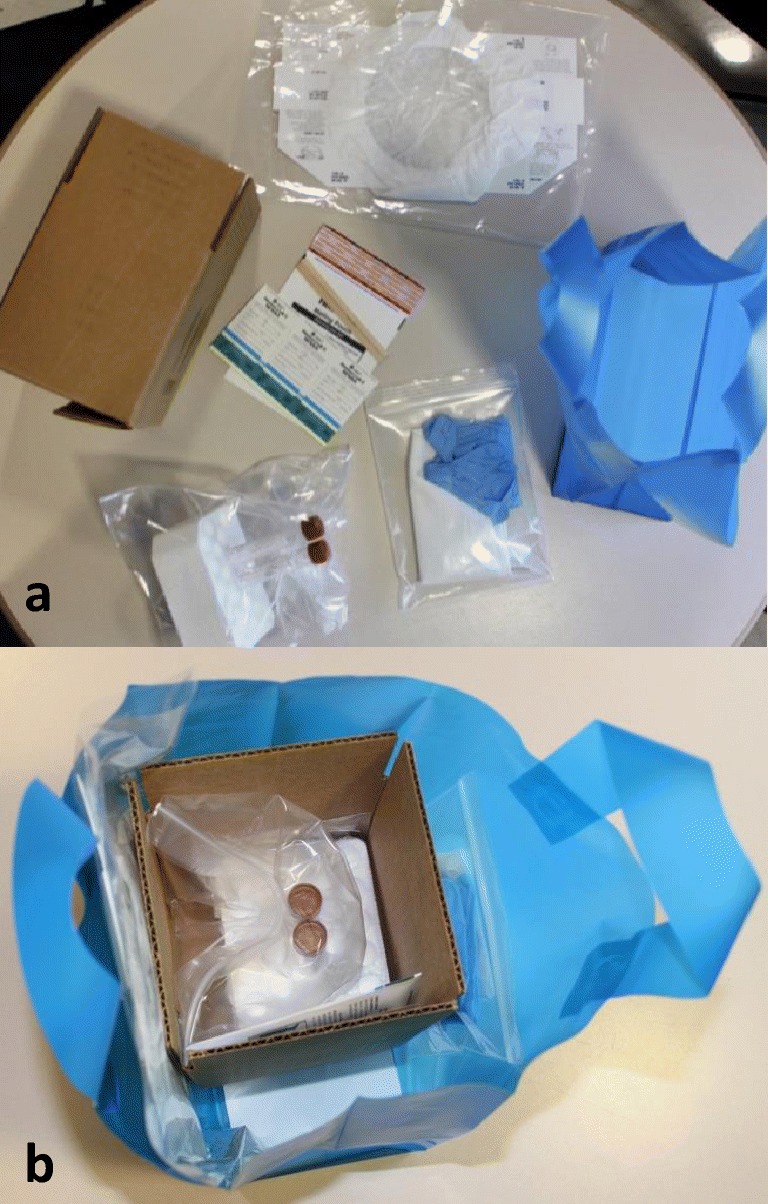
Photographs of the disassembled and assembled at-home stool collection kit. In panel **a**, the three collection media (FOBT card with three collection slides, ethanol, and RNAlater filled tubes) are displayed, along with additional devices, such as gloves and the Protocult collection device, that patients use to collect fecal specimens at home. Panel **b** depicts the completed kit; upon assembly, the kit should resemble the image above. The ethanol and RNAlater tubes should be standing upright. At this stage, the kit is ready for delivery to patients

Place the Styrofoam rack (upright and sealed in a large zip bag) and FOBT card (enclosed in the biohazard envelope) into a cardboard shipping box. Place the box and contents inside a shopping bag. To the shopping bag, add two Protocult collection devices (one is a backup), bag of supplies (gloves, pad, spatulas), and return packaging tape (for shipment via courier). Include an informed consent form, illustrated collection instructions (see Online Resource 2), and other questionnaires (e.g., Bristol stool chart) as desired. The kit should resemble that displayed in Fig. 1a, b.

Using the at-home stool collection kit is easy and safe. The Protocult collection device is attached to the toilet seat and used to collect the stool. The collection device is laid atop the absorbent pad on a sturdy surface. Specimens are individually aliquoted. Using the FOBT card and wooden applicators, a thin layer of stool is smeared onto six windows (two windows under each of three slides). The flaps are closed and the FOBT card secured in the biohazard envelope. Each collection tube has a spoon attached to the cap that is used to collect ~ 1 g of stool; the small spatula is used to level off excess stool. The spoon is returned to the collection tube and the cap is secured tightly. Each tube is shaken gently and placed upright into the Styrofoam rack. The rack and tubes are sealed in the large zip bag. All items are placed into the cardboard box, including paper forms. Specimens are returned to the clinic in person or by courier within 3 days. Upon receipt at the laboratory, each FOBT card slide is labeled with a unique ID and placed in a 4 in. × 6 in. zip bag (4 mil) for storage at − 80 °C. Each tube is labeled with a unique ID, vortexed for 5 s, and archived at − 80 °C; however, if resources are available, stool should be aliquoted into smaller quantities to minimize freeze/thaw cycles during processing.

## Discussion

We describe the assembly and use of a comprehensive yet customizable at-home stool collection kit. Briefly, patients collect one stool sample, preserve specimens using three standardized methods (FOBT card, RNAlater, and 95% ethanol), store specimens at room temperature, and return them to researchers within 3 days. Each preservation method has been extensively evaluated for validity, reproducibility, and stability and is considered optimal for use in studies of the gut microbiome [10–16]: FOBT cards are well-suited for 16S rRNA gene sequencing to determine microbial composition and relative abundance, RNAlater-preserved stool is optimal for metatranscriptomics to determine the functional roles of the microbiota, and 95% ethanol–preserved stool is optimal for metabolomics analyses to identify microbial- and dietary-derived metabolites produced in the gut. The kit was designed to increase compliance in challenging populations, specifically cancer patients struggling with weakness and constipation. The kit can also be used for patients who develop diarrhea, as the Protocult collection device and collection tubes with spoon attachments are suitable for use with loose stool. Room temperature storage eliminates the need to utilize patients’ refrigerators or freezers and transport specimens using heavy ice packs. A 3-day collection and transit window allows for multiple collection attempts in case of constipation.

**Fig. 2:**
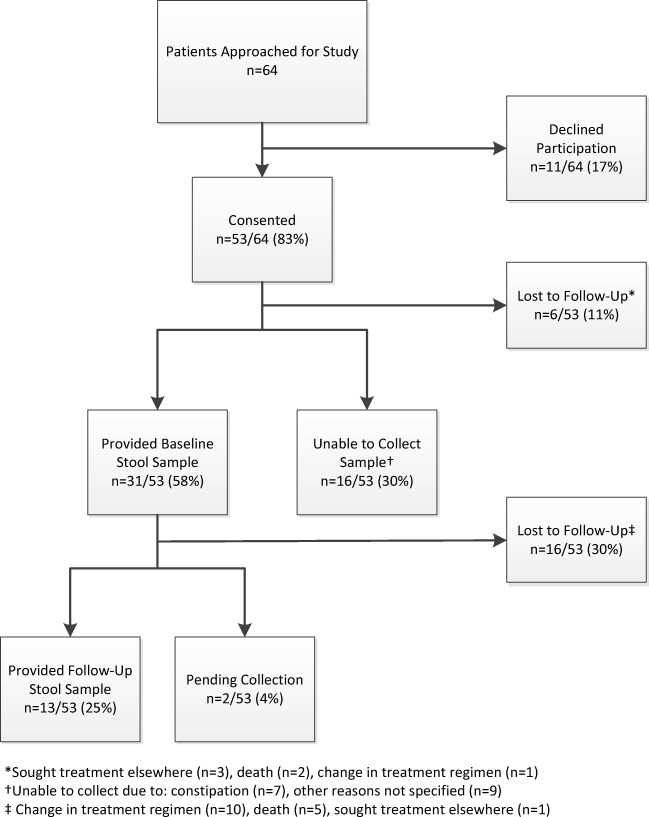
Study participation among late-stage cancer patients in an ongoing, longitudinal study, January 1, 2018—August 31, 2018

In an ongoing, longitudinal gut microbiome study among late-stage lung cancer patients, we have observed that 83% (53/64) of patients agree to participate and 58% (31/53) comply by providing the baseline stool sample (Fig. 2). Reasons for non-compliance at baseline were not systemically collected; however, seven participants communicated that they were unable to collect due to constipation. Twenty-five percent (13/53) of participants provided a stool sample at follow-up (approximately 8 weeks post-baseline), and two were pending collection at the time of manuscript submission.

We hope that by providing an in-depth description and video of this protocol, population scientists and clinicians will be encouraged to add standardized stool sample collection to existing studies. Although the clinical utility of the microbiome has yet to be determined, accumulating evidence demonstrates that the gut microbiome plays a significant role in human health and disease, and certainly warrants further investigation.

## Electronic Supplementary Material

ESM 2 (PDF 14103 kb)
