# Supplementary material for: A Customized At-Home Stool Collection Protocol for Use in Microbiome Studies Conducted in Cancer Patient Populations
Source: Microb Ecol. 2019 Mar 30;78(4):1030–4. doi: 10.1007/s00248-019-01346-2 (PMC6768769; doi:10.1007/s00248-019-01346-2)
Supplement: Supplementary file 2 — (PDF 14103 kb) [file 248_2019_1346_MOESM2_ESM.pdf]

Stool Collection Kit Instructions

Study:
Principal Investigator:

Research Coordinator:
Phone:
E-mail:
Pager:

Copyright © 2018 Moffitt Cancer Center and Research Institute

MATERIALS

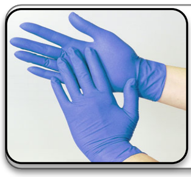

A clean pair of nitrile gloves (safe for latex allergies).

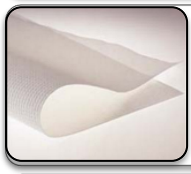

Large absorbent pad to lay on sturdy surface.

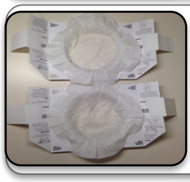

Two disposable water-resistant Protocult™ stool collection pouches with adhesive tape. The second device is to be used as a backup in case urine contamination occurs.

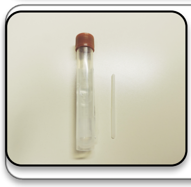

Two pre-labeled plastic tubes with a spoon attached to the lid and plastic spatula. One tube contains 8mL of 95% ethanol and the other contains 8mL of a liquid preservative. All are labeled with your sample information.

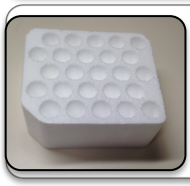

Tube rack to secure the tubes upright.

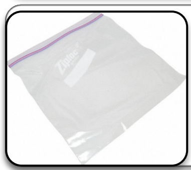

Two zip bags: 1 large bag to hold tubes and rack with small absorbent layer, 1 small bag to hold pad, gloves, and stool spatulas. The small bag can be discarded after use.

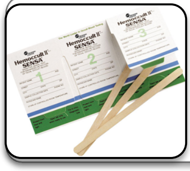

Hemoccult II® SENSA triple slide FOBT card with applicator sticks.

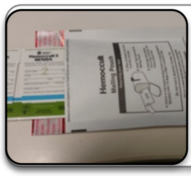

Envelope for Hemoccult® II SENSA triple slide FOBT card.

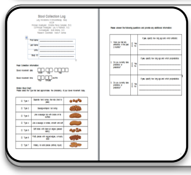

A "Stool Collection Log" to document the time of collection and sample information.

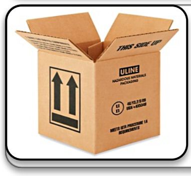

A 5"x5"x6'' shipping box.

Copyright © 2018 Moffitt Cancer Center and Research Institute

INSTRUCTIONS

Samples should be collected within 3 days of your next clinic visit.

1. Remove the tube from the cardboard box and unpack the collection kit. Lay the provided pad on a sturdy surface, such as the sink basin or countertop.
2. **Urinate before stool collection** in order to avoid contaminating your stool sample with urine.
3. Wash your hands with soap and water.
4. Lay one of the Protocult™ stool collection pouches on a flat surface with the instructions facing upwards. Fold the outer flaps upwards. Peel the backing off of the adhesive tape on both sides of the pouch.

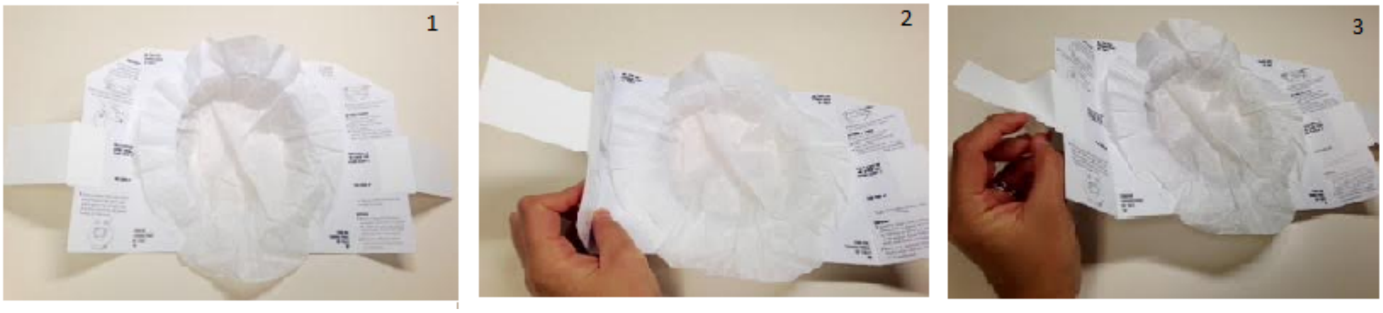

5. Wearing the gloves provided, attach the Protocult™ stool collection pouch over the toilet bowl (avoiding any contact with the toilet water) and stick the tape to the toilet seat.

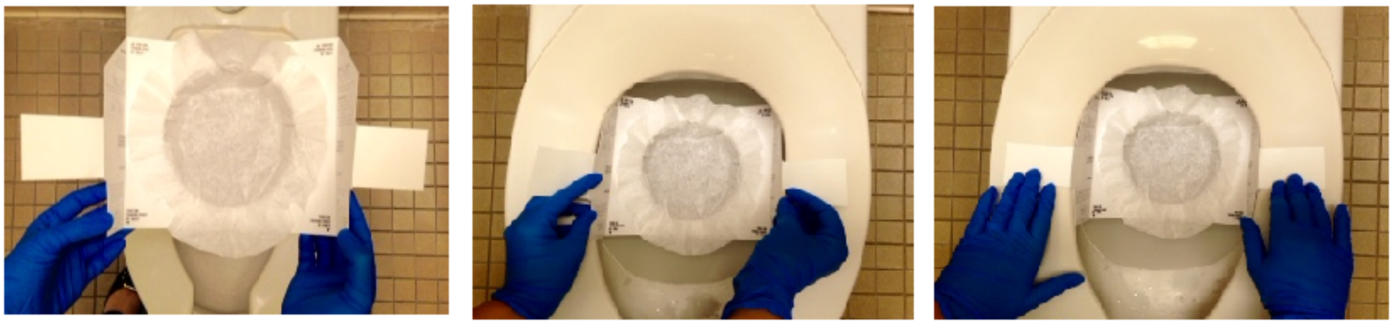

6. Empty your bowels directly into the collection pouch. Please avoid urinating in the collection pouch. **If accidental urination occurs, please discard the sample and the pouch (see step 18), and use the second pouch provided.**

Copyright © 2018 Moffitt Cancer Center and Research Institute

7. After your bowel movement, remove the stool collection pouch from the toilet and lay it on the absorbent pad.

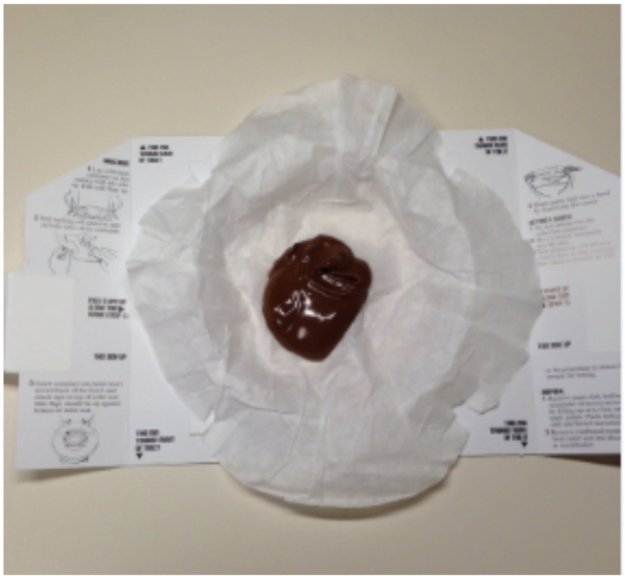

8. Lay the test card flat next to the sample and open the flaps in preparation for the collection.

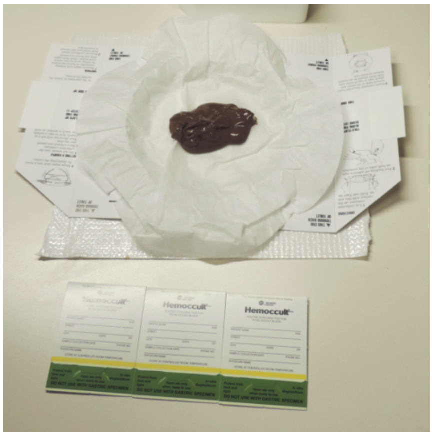

9. Obtain a stool sample with the provided applicator stick and apply a <u>thin</u> smear in box A. Reuse applicator stick to obtain a 2nd sample and apply a <u>thin</u> smear in box B.

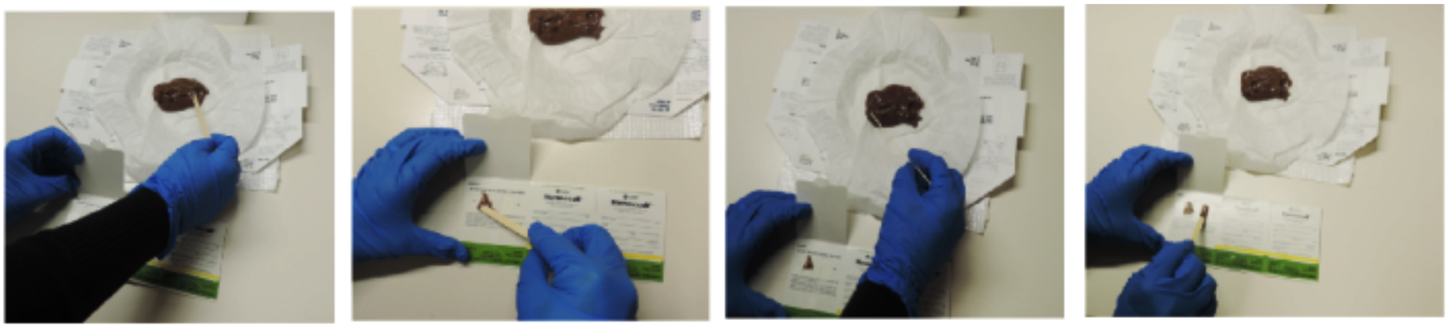

Copyright © 2018 Moffitt Cancer Center and Research Institute

10. Repeat collection with the other 2 slides (step 9) for a total of 6 smears. Please complete the three slides (six windows total).

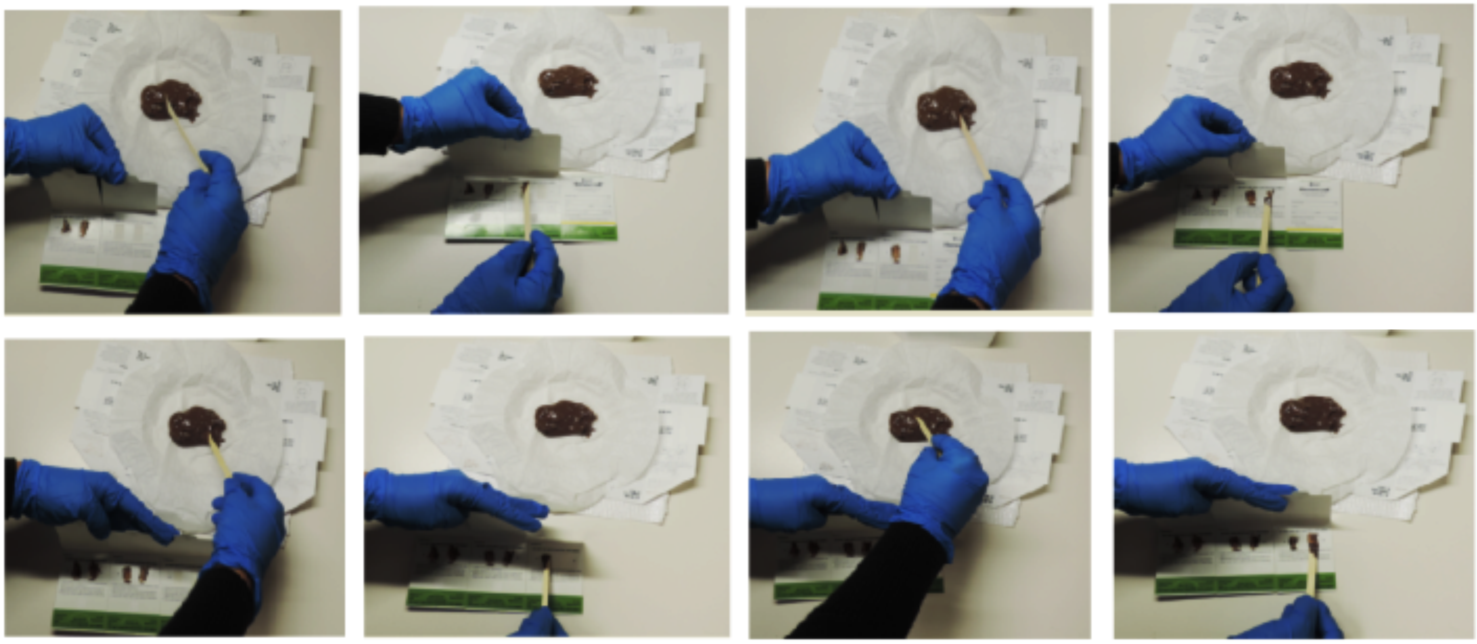

11. Close the flaps and store the test card in the envelope provided.

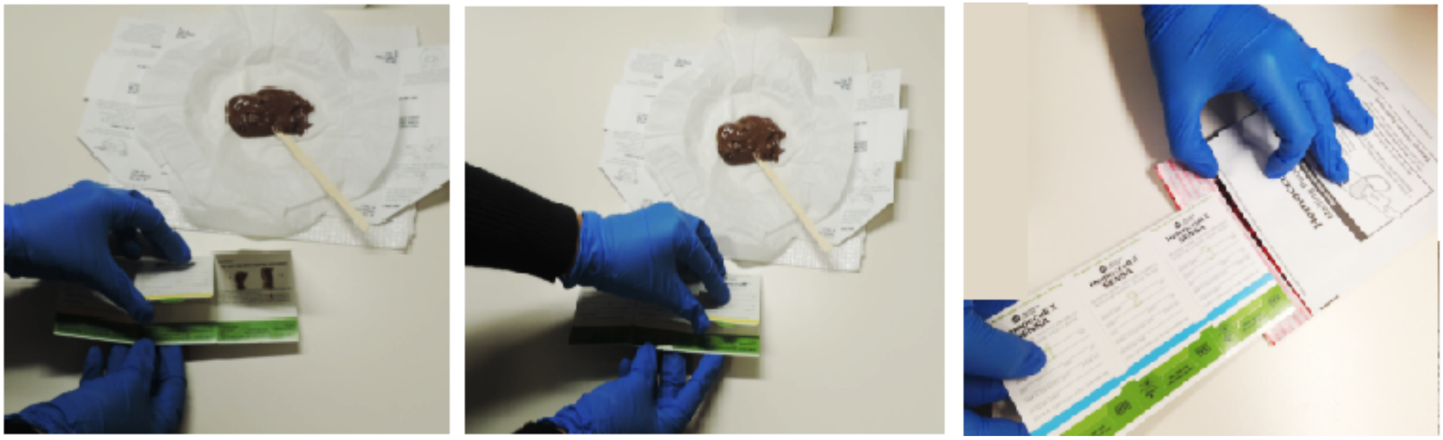

Please **<u>do not</u>** place the sample cards inside the plastic zip bag.

12. Keeping the tube upright, gently twist the cap off – AVOID SPILLING THE LIQUID. If accidental spilling occurs, rinse any surface exposed with water. <u>Do not</u> add water or any other liquid to the tubes.

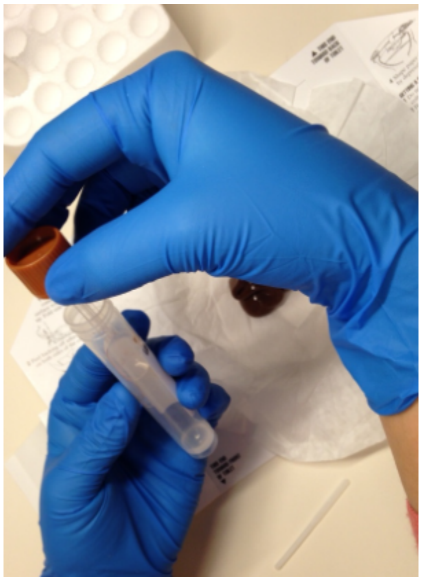

13. Fill the scoop with stool and use the provided plastic spatula to level off the excess – DO NOT OVERFILL THE SPOON OR THE TUBE.

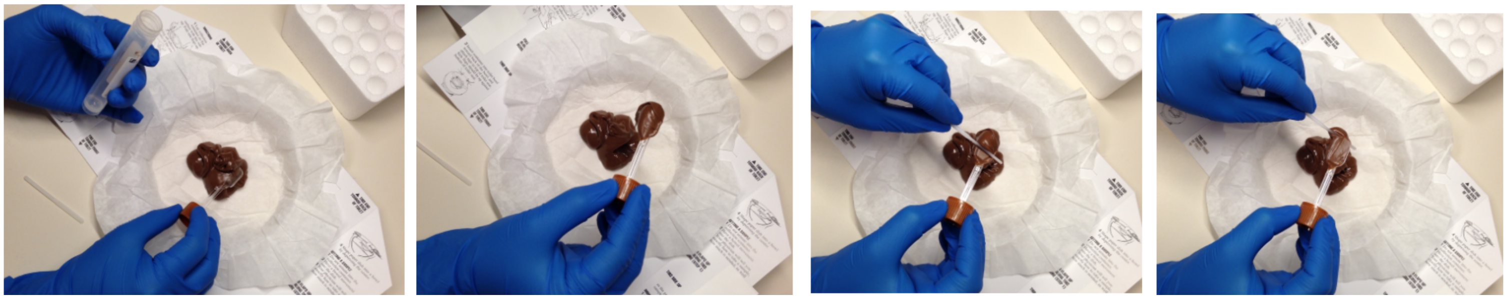

14. Place the feces-filled scoop back into the tube, tighten the lid, gently shake the tube to ensure that the liquid covers the stool sample, and secure the tube (UPRIGHT) in the tube rack.

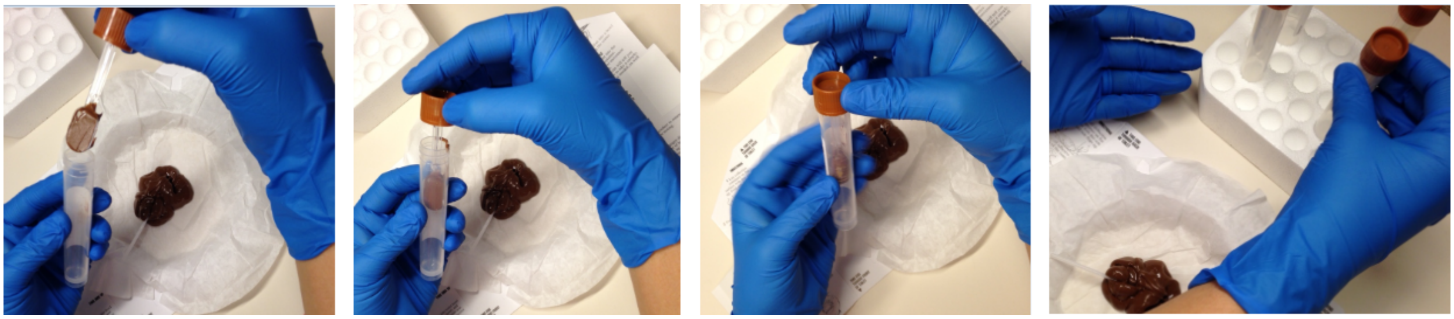

15. Repeat collection with the other tube (steps 12, 13, & 14).

Copyright © 2018 Moffitt Cancer Center and Research Institute

16. Insert the entire tube rack and all plastic tubes inside the large zip bag with the small white absorbent shipping pad, and seal.

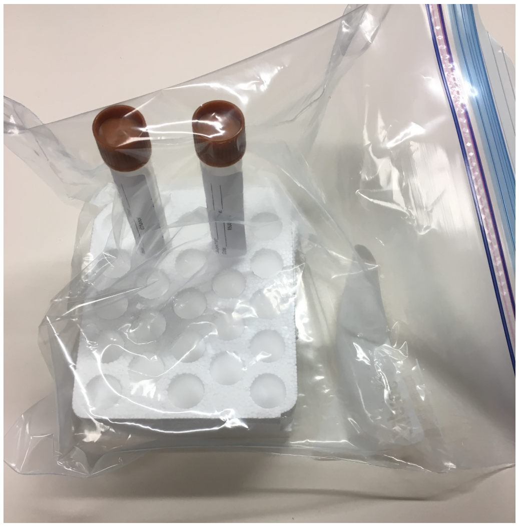

17. Place the sealed zip bag with all tubes inside the cardboard box – KEEP THE TUBES UPRIGHT.

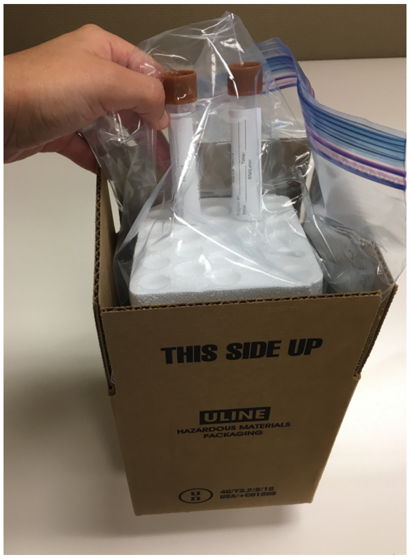

18. The remaining feces can be left in the collection pouch. Detach the centerpiece from the paper frame, and flush the feces and centerpiece down the toilet. The remaining portion of the Protocult™ stool collection pouch can be discarded in the trash.

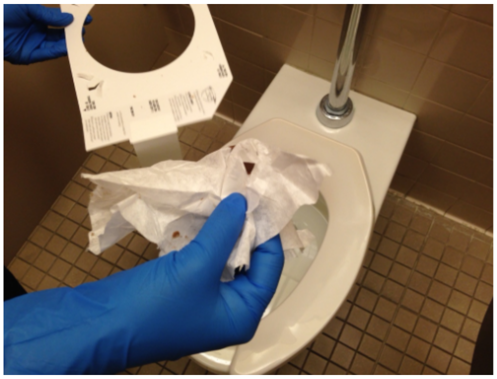

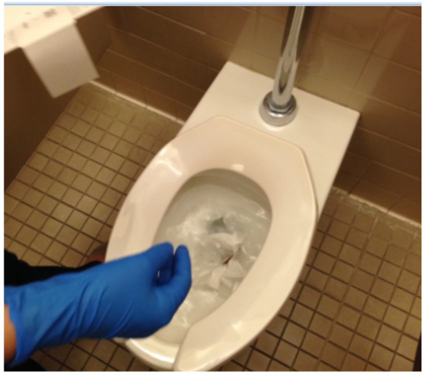

19. Remove your gloves and wash your hands with soap and water.

Copyright © 2018 Moffitt Cancer Center and Research Institute

20. Using the "Stool Collection Log" provided, enter the date and time of collection and describe your stool using chart provided. Fold the "Stool Collection Log".

21. Place the mailing pouch and the "Stool Collection Log" inside of the cardboard box with the zip bag containing the tubes. Keep the box upright at room temperature until you return to the clinic or until shipping.

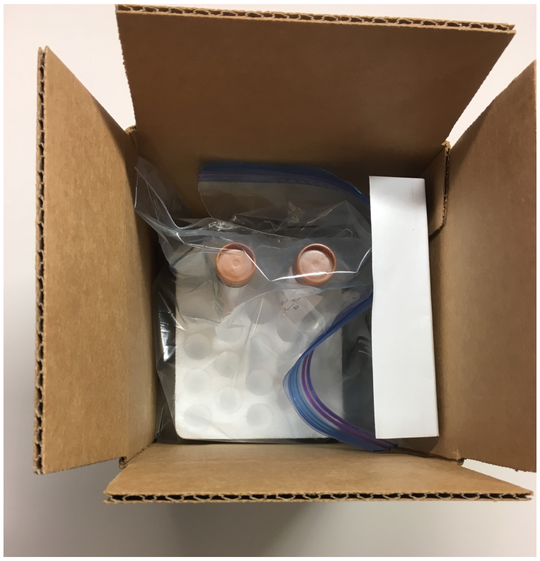

➤ **If** instructed by the Research Coordinator to ship the specimens via courier, please follow these instructions:

1. Complete the collection instructions.

2. Using the tape provided, seal the middle of the box and both sides. Place the pre-paid shipping label on the top of the box. Keep the box at room temperature until the scheduled courier pick-up time.

3. The Research Coordinator may call you to arrange the package pick-up time. Please make sure this time is convenient for you.

➤ If you have any questions or issues please contact the Research Coordinator:

Name:
Phone:
Email:
Pager:

Copyright © 2018 Moffitt Cancer Center and Research Institute
